# Measles epidemic in Southern Vietnam: an age-stratified spatio-temporal model for infectious disease counts

**DOI:** 10.1017/S0950268822001431

**Published:** 2022-09-12

**Authors:** Thi Huyen Trang Nguyen, Christel Faes, Niel Hens

**Affiliations:** 1 Hasselt University, Hasselt, Belgium; 2 The Pasteur Institute, Ho Chi Minh City, Vietnam; 3 The University of Antwerp, Antwerp, Belgium

**Keywords:** Measles, social contact, spatio-temporal analysis, Vietnam

## Abstract

Measles resurged in Vietnam between 2018 and 2020, especially in the Southern region. The proportion of children with measles infection showed quite some variation at the provincial level. We applied a spatio-temporal endemic–epidemic modelling framework for age-stratified infectious disease counts using measles surveillance data collected in Southern Vietnam between 1 January 2018 and 30 June 2020. We found that disease transmission within age groups was greatest in young children aged 0–4 years whereas a relatively high between-group transmission was observed in older age groups (5–14 years, 15–24 years and 25+ years groups). At the provincial level, spatial transmission followed an age-dependent distance decay with measles spread mainly depending on local and neighbouring transmission. Our study helped to clarify the measles transmission dynamics in a more detailed fashion with respect to age strata, time and space. Findings from this study may help determine proper strategies in measles outbreak control including promotion of age-targeted intervention programmes in specific areas.

## Introduction

Measles is one of the most contagious viral diseases. The global reduction of measles incidence during 2000–2016 is a victory of intensive public health efforts, especially in surveillance and measles immunisation activities. There are no animal reservoirs and infections do not result in persistent shedding of the pathogen. Thus, measles transmission can only be sustained and outbreaks can only occur when the susceptible population has attained sufficient size [1]. Between 2016 and 2019, the world experienced a resurgence of measles, with an increase of 556% in the reported incidence observed globally, challenging the feasibility of the disease elimination goal [2]. This is largely due to the replenishment of individuals who are not immunised over successive birth cohorts [3]. In other words, failure in maintaining high levels of measles immunity via vaccination eventually results in an accumulation of susceptibility in the population and an introduction of the virus could facilitate widespread transmission. Although effective vaccination programmes would reduce the supply of susceptible individuals into the population, spatial heterogeneity of the vaccination coverage has been associated with the occurrence of local outbreaks [4, 5]. The circulation of measles virus is also correlated with other stochastic local determinants such as population density, inter-regional human movement [6, 7] and mixing behaviour [8–10]. For example, short-term migration of susceptible populations (e.g. rural-to-urban) may increase the input of susceptibilities in metropolitan areas, and thus exacerbate the risk of outbreaks in these localities [6, 7]. Furthermore, as contact patterns of age-assortative mixing are usually observed [11, 12], the risk of measles infection is greater when individuals in under-immunised communities preferentially cluster in their age class [13, 14].

In Vietnam, the vaccine schedule is a two-dose schedule that the first and second doses of measles containing vaccine (MCV1 and MCV2) are given at 9 and 18 months old, respectively. Despite the high coverage over the last decade (>95% for MCV1 and >80% for MCV2 [15]), sustained epidemics of measles occurred during 2018–2020. The Southern region, which consists of 20 provincial units (the province of Lam Dong, five provinces and a city in the South East region, 12 provinces and a city in the Mekong River Delta region), was heavily affected with more than 26 000 cases reported in total. Heterogeneous distribution of measles incidence in different age groups in relation to space and time was observed in the reported case data. This raised the interest to investigate the spreading patterns of measles in Southern Vietnam and the degree to which these transmission patterns are influenced by age and geographical areas. We fitted an age-structured spatio-temporal statistical model for infectious disease counts [16, 17] in which the number of infections is additively decomposed into an endemic and epidemic component. While the latter component describes the occasional outbreaks that are linked to previous cases within- or between-geographical units, the endemic part captures exogenous factors that explain incidence not directly linked to past counts. To simultaneously investigate the transmission across age groups, we accounted for the age-structured mixing pattern, which was adapted from a social contact study in Vietnam in 2007 [11]. We used routine surveillance data for daily reported counts of measles collected between 1 January 2018 and 30 June 2020 stratified by age group and by provincial unit in the South of Vietnam. Results from this study can provide more insights into the measles outbreak dynamics and more effectively inform age-targeted containment measures in the country (e.g. age-targeted vaccination campaigns, health communications).

## Method

### Measles surveillance data

**Fig. 1.**
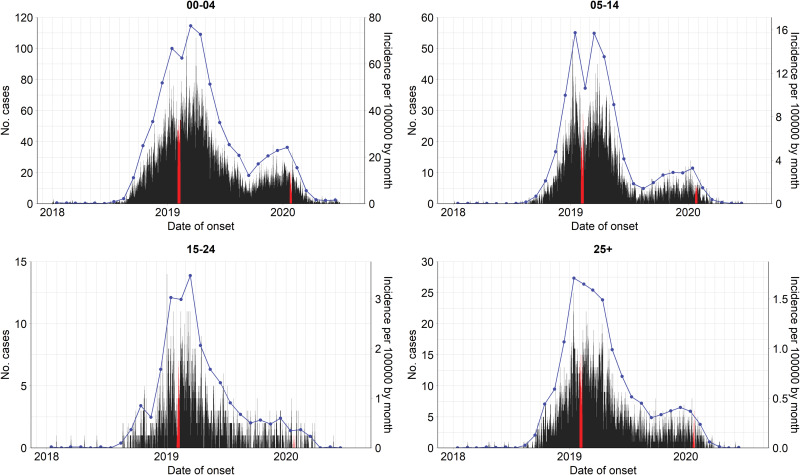
Evolution of age-specific measles cases by date of symptom onset (left axes) and incidence per 100 000 population by month (blue lines, right axes) in Southern Vietnam, 1 January 2018 to 30 June 2020. Lunar New Year in 2019 and 2020 are highlighted in red.

**Fig. 2.**
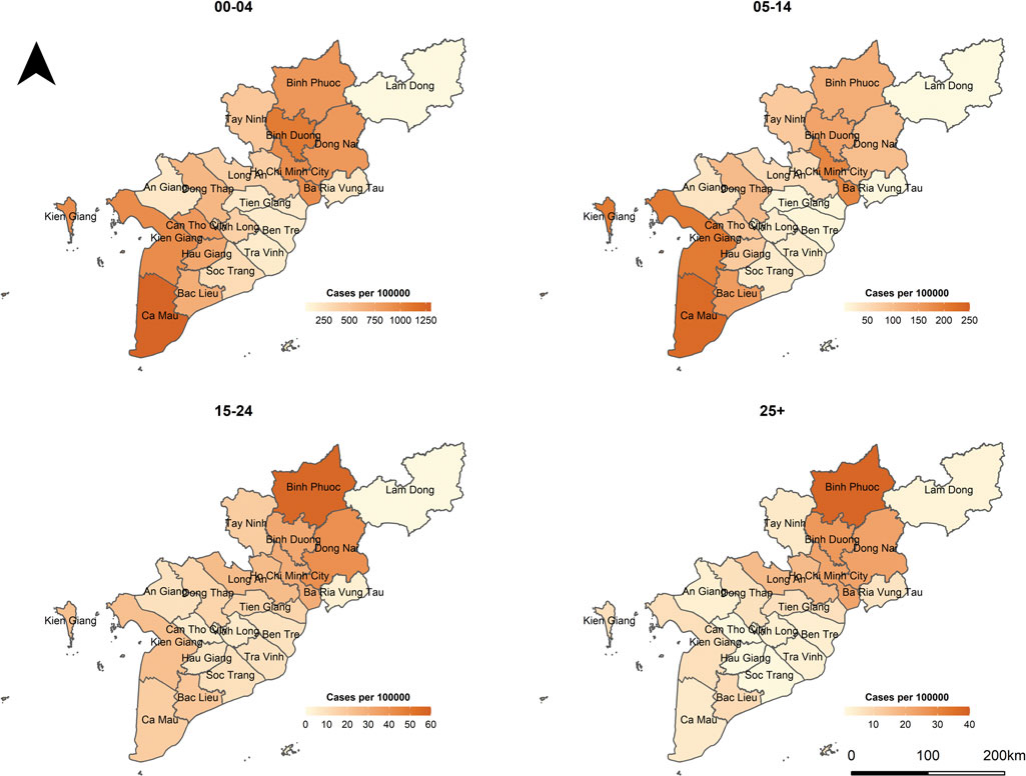
Maps of age-specific cumulative incidence per 100 000 population in Southern Vietnam, 1 January 2018 to 30 June 2020.

In Vietnam, measles is one of the 20 infectious diseases that are required to be notified online to the Electronic Communicable Disease Surveillance System (ECDS) within 24 h post clinical diagnosis. In the case report form, several fields including demographic characteristics, date of illness onset, date of hospitalisation or medical examination of in- and out-patients are obligated to be reported. More information about the disease surveillance and reporting system in Vietnam can be found in [18, 19]. In this study, we analysed measles cases reported between 1 January 2018 and 30 June 2020 in the Southern region. On 11 October 2020, 26 047 individual cases were extracted from the ECDS, accessed by the Pasteur Institute in Ho Chi Minh City, the public health institute that manages the disease surveillance system in the South. For the age-structured spatio-temporal analysis of the outbreak, we categorised individuals into four age groups: 0–4 (children), 5–14 (school children), 15–24 (adolescent and young adults) and 25+ (adults) years of age. Overall, the median age at disease onset was 3 years, ranging from <1 year to 84 years. The most affected age group was 0–4 years, which accounted for 61.4% of measles cases while the 5–14 years, 15–24 years and 25+ years groups represented 22.0%, 4.5% and 12.1% of the total cases, respectively. Based on the date of onset, we aggregated the daily number of cases by age group in each province in which the cases resided. Figure 1 depicts the evolution of daily counts of measles infections and monthly incidence per 100 000 population by age group and Figure 2 presents maps of the age-specific cumulative incidence per 100 000 individuals across all provinces.

### Population data

Age- and province-specific population data were obtained from the census data in 2019 [20]. The Southern population was approximately 36 million in 2019, and in each age group, the population fraction was 6.7% for 0–4 years, 14.9% for 5–14 years, 14.3% for 15–24 years and 64.2% for 25+ years. We assumed that the total population was constant over the period 2018–2020.

### Social contact data

**Fig. 3.**
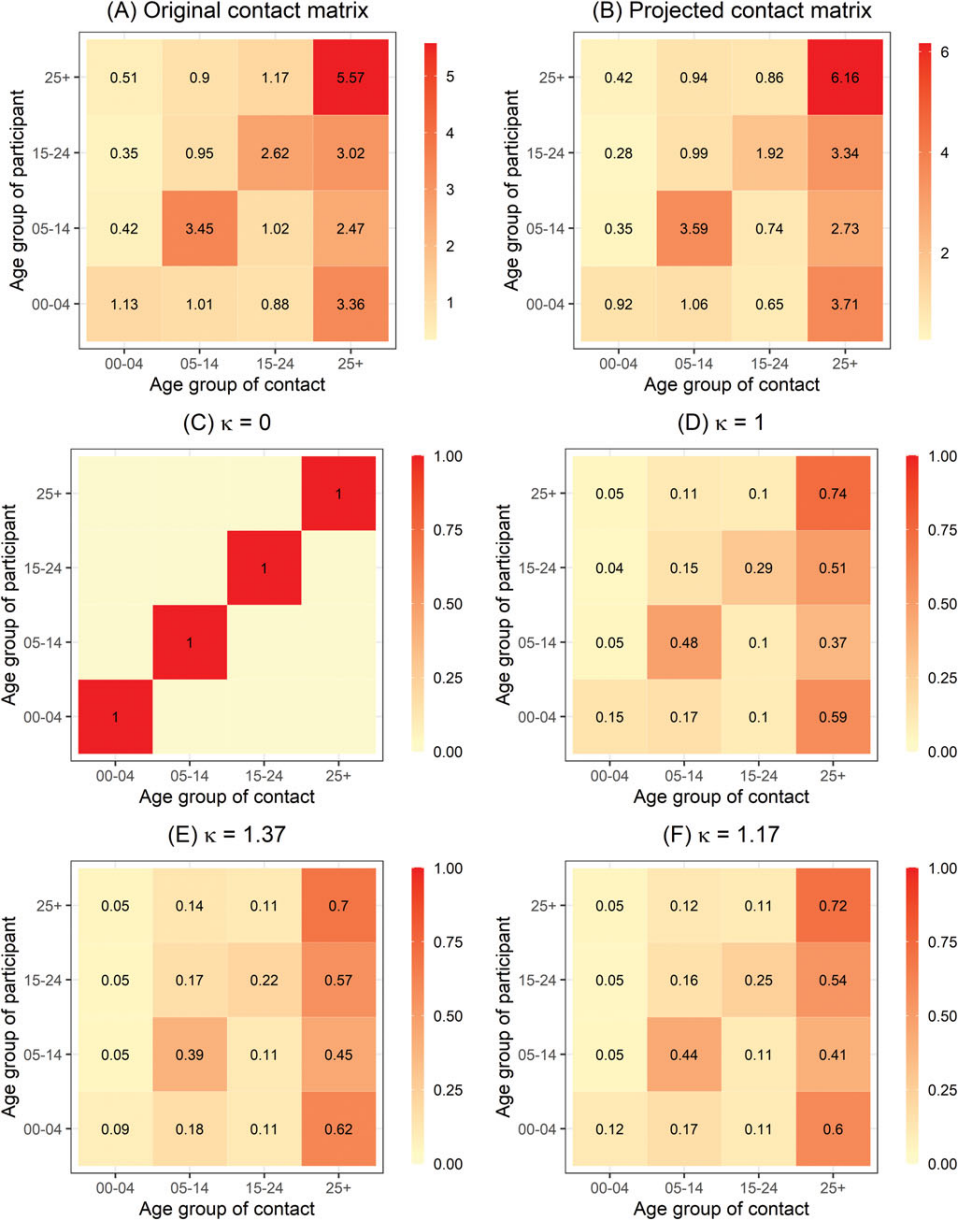
(a) Original age-structured contact matrix ***C*** estimated in Northern Vietnam anno 2007 aggregated to the age groups of interest and (b) the age-structured contact matrix projected for Southern Vietnam ***C***_(***P***)_ based on (a). The entries contain the mean number of contacts made by one participant per day. (c), (d), (e) and (f) refer to the power transformation of row-normalised contact matrix ***C***_(***P***)_ for different values of *κ*.

To reflect the amount of mixing between age groups, we used social contact data, adapted from an empirical contact matrix in a survey in the Red River Delta region of Northern Vietnam in 2007 [11]. The matrix was transformed because of the difference in demographic structures between Northern and Southern Vietnam and hence, directly using the original contact matrix would not be valid in our study. First, we extracted the social contact patterns aggregated to the age groups of interest from the Social Contact Rates (SOCRATES) Data Tool (http://www.socialcontactdata.org/socrates/) [21]. This age-structured contact matrix 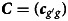 provided the average non-negative number of contacts of a person in age group *g*^′^ (rows) with a contact in age group *g* (columns) in 1 day (Fig. 3a) aggregated over weekdays or weekends, contact duration, physical or non-physical contacts and gender. Next, we projected the social contact matrix for Southern Vietnam 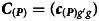 using the density correction method proposed by Arregui *et al*. [22]. The projected contact matrix is a product of an intrinsic connectivity matrix ***C***(*N*/*N*_*g*_) and the fraction of individuals in the age group of the contact 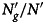, where *N*_*g*_ and 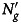 are the demographic structures in 2009 (Red River Delta region) and 2019 (Southern region), respectively. Note that because the age-structured population numbers of Red River Delta region are not available for 2007, we used population of Red River Delta region from the 2009 census [20]. The obtained contact matrix is shown in Figure 3b.

### Age-structured spatio-temporal analysis

In general, we leveraged an endemic–epidemic modelling framework for multivariate infectious disease counts first introduced by Held *et al*. [16] and extended in a series of publications [5, 17, 23–26, 38, 39]. The framework subsequently incorporated the age-structured contact matrix (possibly adjusted) to better understand disease spread in the scenario of heterogeneous mixing [17].

Formally, let *Y*_*grt*_ denote the number of cases in age group *g* = 1, …, *G* in province *r* = 1, …, *R* at time *t* = 1, …, *T*. Conditional on the number of cases at the previous time point *t* − 1, the counts are assumed to follow a negative binomial distribution with conditional mean *μ*_*grt*_:1
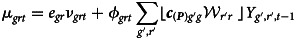
and a variance *μ*_*grt*_(1 + *μ*_*grt*_*ψ*_*g*_) with a group-specific overdispersion parameter *ψ*_*g*_ > 0 [17, 26]. Note that if *ψ*_*g*_ = 0, the distribution simplifies to the Poisson distribution. The mean *μ*_*grt*_ is decomposed into endemic and epidemic components. The former component exhibits baseline patterns. The latter component involves an autoregressive effect that links cases at time point *t* in unit *r* with observations at the previous time point *t* − 1 and in units *r*^′^ = 1, …, *R*. Specifically, the non-negative parameters *ν*_*grt*_ and *ϕ*_*grt*_ are modelled as log-linear predictors:2
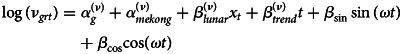
3
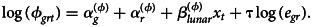


The above two equations contain age-specific fixed effects (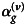, 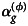). Because we deemed that fewer cases were reported during the Lunar New Year, we included an indicator for the holiday in 2019 and 2020 with coefficient *β*_*lunar*_ (*x*_*t*_ = 1 for dates from 2 to 10 February 2019 and from 23 to 29 January 2020, otherwise *x*_*t*_ = 0). In the endemic component, to adjust for the possibly different number of individuals at risk in each province and age group, we included the population size *e*_*gr*_ as an offset [25]. To allow for the differences amongst the two main administrative regions, 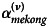 is included to resemble the effect of the Mekong River Delta region. We also assumed that the disease incidence varies with a linear time effect (*β*_*trend*_) and an overall seasonal sine–cosine term where the sinusoidal wave of frequency *ω* identified as 2*π*/365 for daily continuous measurement [24]. In the epidemic component, we allowed for province-specific effects 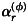 and accounted for population size *e*_*gr*_ to quantify how ‘attraction’ to a province *r* scales with population size in group *g*, in which the strength of population scaling factor *τ* is to be estimated [25, 27].

To determine transmission weights from age stratum *g*^′^ to age stratum *g* (i.e. 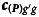), and from area *r*^′^ to *r* (i.e. 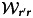), the product 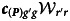, which is row-normalised, i.e. 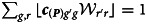 was introduced in the epidemic component. In ideal circumstances, to best reflect the transmission between strata, the matrices of contact and mobility should be displayed by age and province. Nevertheless, such data sources are not easily available as collecting contact and movement patterns is cumbersome. In our study, we took the overall estimate of contact data for age-group weights and used the power law approximation for the spatial weights. First, the age-group weights 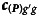 are row-normalised and then raise it to the power *κ* ≥ 0, i.e. 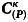 [17]. In an easy interpretation, the limit *κ* = 0 corresponds to no mixing between different age groups, i.e. the diagonal contact matrix ***C***_(***P***)_ = ***I*** (Fig. 3c). When *κ* = 1, the contact matrix represents the given projected contact matrix (Fig. 3d). As *κ* → ∞, the transmission from an infected person to any individual of any age group has the same distribution with other groups regardless of the group they are in [17]. We also consider homogenous mixing scenario in the epidemic component. Second, the non-negative weight 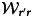 in the epidemic component describes the strength of transmission between geographical units. In the absence of mobility data, it can be estimated using a power law formulation in terms of adjacency order 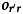, which is a discrete distance measure of neighbourhood order between unit *r*^′^ and *r* [25]. The power law weights 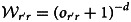, where *d* > 0 is the decay parameter to be estimated, thus give unit weight to local transmission when *r*^′^ =  *r* and then decay to promote the spatial transmission from unit *r*^′^ to unit *r*. The power law weights can be age-dependent (replacing *d* by 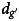) [17]. In this study, 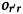 ranges from 0 to 7.

All procedures were performed using R software version 4.0.5, packages surveillance version 1.19.1 [28] and hhh4contacts version 0.13.1 [17]. In each model, maximum likelihood estimates of parameters and 95% confidence intervals (95% CIs) were obtained numerically. Model selection is performed according to the smallest Akaike information criterion (AIC) value.

### Sensitivity analysis

We performed a sensitivity analysis using weekly aggregation of the surveillance data. We also ran another sensitivity analysis to assess the impact of different forms of contact matrix on our results, including the original contact matrix, and the per capita contact rates (i.e. dividing the mean number of contacts per day per participant in group *g*^′^ to the Vietnamese population size in 2009 and in 2019 [20] in contact group *g*).

### Ethical consideration

As part of public health surveillance system in Vietnam, case-based data of measles were routinely collected for disease control purposes. Anonymised data, i.e. without identification of patient information, were provided for use in this study. Therefore, this study did not require ethical approval.

## Results

**Fig. 4.**
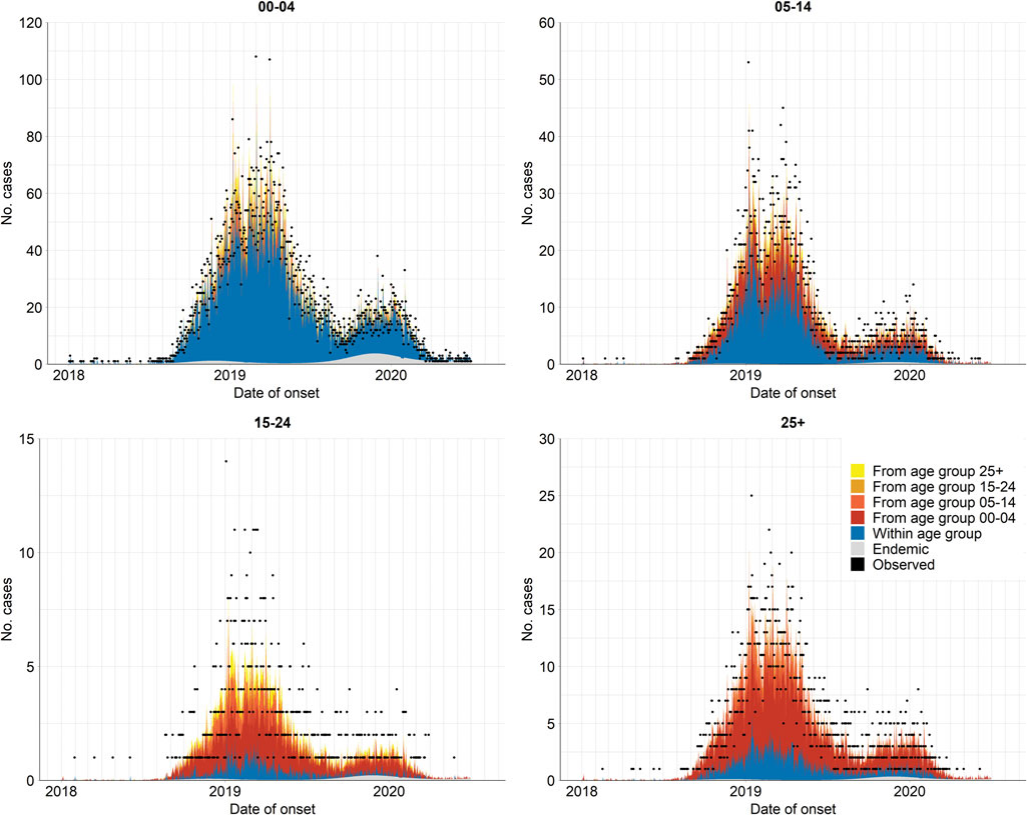
Fitted components of the selected model (i.e. model using the projected contact matrix ***C***_(***P***)_ and assumption of age-specific power law), aggregated by age group over all provinces. The dots indicate the observed number of daily infections.

Table 1 summarises the age-stratified spatio-temporal models with respect to different assumptions of age-structured contact matrix and spatial transmission weights. Because the AIC values of two models with age-dependent power law (two last rows) are not largely different given the large sample size, we select the simplest model that incorporates the projected matrix ***C***_(***P***)_ (the second-to-last row) for further exploration. Coefficient estimates of the selected model are presented in Table 2. Overall, the disease transmission was dominated by transmission within age groups, which contributed to 59.9% of measles cases while the contribution of transmission between age groups was 35.5% of cases. The endemic component added the remaining 4.6% of total cases (Supplementary Fig. S1). The transmission within and between age strata are described in Table 3 and visualised in Figure 4. In the youngest age group, 74.9% of the disease cases were predominantly affected by within-age-group transmission whereas only 19.1% of the cases were explained by transmission from other age groups. In those aged 5–14 years, the contribution of within- and between-age-group transmission seemed balanced with 49.1% and 49.2% of disease incidence, respectively. Interestingly, as opposed to the spreading pattern of measles in the 0–4 years group, we found a large number of cases in the 15–24 years and 25+ years groups attributable to transmission from other age groups. Specifically, an estimated 48.1% of cases aged 15–24 years and 57.6% of cases aged 25+ years were infected by age group 0–4 years, respectively, whereas transmission within age groups contributed to 13.1% of cases aged 15–24 years and 19.2% of cases aged 25+ years.

**Table 1.** Summary of age-stratified spatio-temporal models for surveillance data of measles in Southern Vietnam

Model with assumption of					
Spatial structure	Contact matrix	Number of parameters	Log-likelihood	AIC	The power adjustment *κ* of the *C*_(*P*)_ (95% CI)
Purely endemic model		13	−39 986	79 999	–
Power law (all age groups)	No mixing (***C***_(***P***)_ = ***I***)	39	−30 430	60 939	–
	Homogeneous mixing (***C***_(***P***)_ = 1)	39	−29 867	59 813	–
	Projected contact matrix ***C***_(***P***)_	39	−29 805	59 689	–
	Adjusted contact matrix 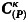	40	−29 794	59 668	1.37 (1.22–1.53)
Group-specific power law	Projected contact matrix ***C***_(***P***)_	42	−29 748	59 580	–
	Adjusted contact matrix 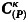	43	−29 743	59 573	1.17 (1.06–1.30)

The first two columns list the fitted models corresponding to different assumptions on spatial transmission weights and the projected age-structured contact matrix ***C***_(***P***)_. The endemic-only model in the first row contains the endemic component only.

**Table 2.** Estimated parameters, their 95% CIs and standard errors in the selected model (model with age-specific power law and projected contact matrix)

	Estimate	Standard error	CI 2.5%	CI 97.5%
Endemic component				
Intercept	−16.653	0.186	−17.018	−16.287
Group 5–14	−3.135	0.272	−3.668	−2.601
Group 15–24	−3.587	0.257	−4.091	−3.082
Group 25+	−4.696	0.244	−5.174	−4.218
Mekong group	0.463	0.137	0.196	0.731
Trend *t*	0.003	2.2 × 10^−4^	0.003	0.003
Lunar New Year	−0.181	0.595	−1.348	0.985
Sin term	−0.684	0.086	−0.853	−0.516
Cos term	0.641	0.089	0.467	0.815
Epidemic component				
Intercept	−6.276	1.217	−8.661	−3.892
Group 5–14	−2.224	0.086	−2.393	−2.055
Group 15–24	−3.105	0.094	−3.289	−2.921
Group 25+	−4.404	0.237	−4.869	−3.940
Ho Chi Minh City	2.448	0.221	2.016	2.881
Binh Phuoc	2.425	0.137	2.156	2.694
Tay Ninh	1.536	0.142	1.257	1.815
Binh Duong	2.425	0.141	2.148	2.701
Dong Nai	2.363	0.156	2.057	2.669
Ba Ria Vung Tau	1.154	0.149	0.863	1.445
Long An	1.744	0.137	1.476	2.013
Dong Thap	2.195	0.136	1.928	2.462
An Giang	1.497	0.145	1.213	1.782
Tien Giang	0.946	0.146	0.661	1.231
Vinh Long	1.446	0.155	1.142	1.750
Ben Tre	1.063	0.159	0.751	1.375
Kien Giang	2.635	0.135	2.369	2.900
Can Tho City	2.263	0.142	1.985	2.540
Hau Giang	2.049	0.163	1.731	2.368
Tra Vinh	1.348	0.162	1.031	1.665
Soc Trang	1.524	0.151	1.228	1.820
Bac Lieu	2.353	0.152	2.055	2.652
Ca Mau	2.772	0.138	2.502	3.042
The power of the population scaling factor	0.486	0.105	0.281	0.691
Lunar New Year	−0.064	0.051	−0.163	0.036
Decay *d*_00−04_	1.829	0.055	1.725	1.941
Decay *d*_05−14_	2.769	0.114	2.555	3.000
Decay *d*_15−24_	2.991	0.325	2.418	3.700
Decay *d*_25+_	3.295	0.231	2.872	3.781
Overdispersion				
*ψ*_00−04_	0.171	0.012	0.147	0.195
*ψ*_05−14_	0.306	0.029	0.249	0.364
*ψ*_15−24_	0.227	0.073	0.083	0.370
*ψ*_25+_	0.153	0.033	0.088	0.218

**Table 3.** Proportion (%) of cumulative measles cases estimated from the selected model that are attributable to endemic, within age group and from other age group transmission

Infectee age group	Endemic component	Epidemic component in infector age group			
		0–4	5–14	15–24	25+
0–4	6.0	74.9	10.4	2.0	6.7
5–14	1.7	39.1	49.1	3.3	6.8
15–24	4.8	48.1	20.8	13.1	13.2
25+	2.7	57.6	15.8	4.7	19.2

**Fig. 5.**
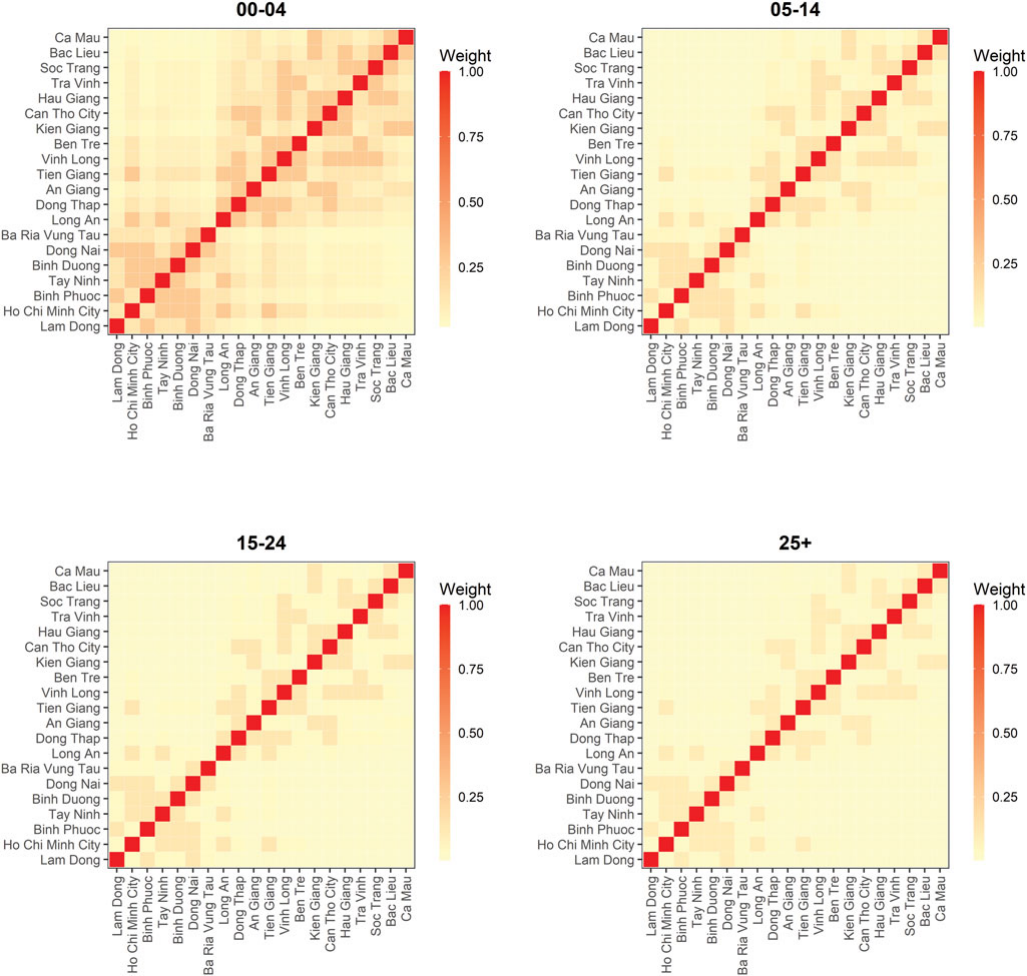
Estimated spatial transmission weights between provinces by age group.

When scaling the epidemic component with the population size, we found that the attraction to province *r* scaled slower than proportional with population size of age group *g*. The corresponding power of the population scaling factor was estimated at *τ* = 0.49 (95% CI 0.28–0.69). Moreover, the spatial diffusion of the disease across provinces followed the age-dependent power laws (Fig. 5). The group-specific decay parameter 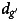 increases from 1.83 (95% CI 1.73–1.94) in the youngest to 3.30 (95% CI 2.87–3.78) in the oldest age groups, meaning that a stronger decay of transmission was observed for more distant provinces in older age groups. In other words, the spatial interaction of nearby provinces is more important to capture the dynamics of measles spread across age strata.

The overdispersion parameters corresponding to the counts in age groups 0–4 years, 5–14 years, 15–24 years and 25+ years groups in the selected model were estimated at 0.17 (95% CI 0.15–0.20), 0.31 (95% CI 0.25–0.36), 0.23 (95% CI 0.08–0.37) and 0.15 (95% CI 0.09–0.22), respectively. This implies that the assumption of *Y*_*grt*_ following the negative binomial distribution is more suitable in the model than the Poisson distribution (*ψ*_*g*_ = 0).

Results of the sensitivity analyses are presented in Supplementary Table S1. We found that the results are robust when applying to different contact matrices (the original and contact rate matrices) in the model. When we changed the resolution of data into weekly intervals (but keeping the projected contact matrix), the lowest AIC value was observed in the model with power-adjusted projected contact matrix and age-specific power law. The estimated power adjustment *κ* of this model was low (0.16; 95% CI 0.11–0.22), meaning that the transmission within age group in the epidemic component summarises more information than suggested in the projected contact matrix (Supplementary Figs S3B and S4). Nevertheless, without power adjustment of the (projected) contact matrix, the weekly aggregation resulted in a contribution of transmission of within and between age groups, similar to that of the main findings (Supplementary Fig. S5).

## Discussion

Using a regression-oriented, endemic–epidemic time series model, we performed a detailed analysis of the transmission dynamics of measles outbreak with respect to age strata, time and space in Southern Vietnam during 2018–2020. Overall, the transmission of measles in the outbreak was built by the intricate reciprocity between different age strata across geographical regions.

It is apparent that measles is a childhood disease. Sixty-one per cent of the infections in the 0–4 years group indicated immunity gaps among these cohorts. This could be because they were not fully covered by two doses of MCV, which are currently administered at 9 and 18 months of age in Vietnam. Studies on the most recent outbreak in Vietnam (i.e. the 2013–2014 outbreak) indicated a high proportion of young children not accomplished two doses of MCV [29] or had an insufficient level of protection against measles infection [30]. Moreover, measles antibody levels in children vaccinated the first single dose, especially those received at 9–11 months, demonstrated a failure to induce an adequate effective immune response [31]. This highlights the importance of compliance of two vaccine doses in age-eligible children to prevent them contracting measles. In addition, we must not overlook a substantial proportion of cases in older children, adolescents and adults. Although a stabilisation of the seroprevalence level until 10 years of age and a fluctuating seroprevalence level in people aged 10–20 years were observed, there were large discrepancies in seroprevalence level at the spatial scale, for example in Ho Chi Minh City a particularly low seroprevalence level in 16–17 years old was observed [30]. Studies in China confirmed a significant decrease of seropositivity over time after vaccination in those aged from 6 to 14 years [32] and a lower sero-protection level in the 15–19 compared to 5–9 years groups [33]. However, older children and adolescents in our study could be unvaccinated in the national supplementary immunisation campaigns implemented in Vietnam in 2014–2015 although these activities targeted those aged 1–14 years (cohorts 2000–2013). Likewise, we suspected that adult cases, especially those aged 25–35 (accounted for 9.4% of total cases, data not shown) likely remained vulnerable because of missed vaccination during the introduction of measles immunisation programme (1983–1989) [34] rather than the result of waning immunity. Further evaluation on the age-specific immunity profile for measles is necessary. The need for supplementary immunisation strategies targeted in adolescents and young adults should be also considered to reduce the residual susceptibility in these populations.

Our study once again confirmed that a model incorporating social contact data has projected the infectious disease dynamics better than those with assumption of random mixing [10, 12, 13]. From the model fitted, we were able to quantify the amount of within-group transmission and effectively capture ‘who acquired infection from whom’. This model helped to explain the measles transmission mechanism across age groups. Because of the age-assortative pattern of contact rates [11], an infected individual is more likely to transmit the disease to a susceptible person in their same age group. The increased within-group interaction amongst children aged 0–4 years increases the risk of infection in this group and thus may act as a driving factor of the outbreak. In addition, the role of school-aged children in facilitating disease spread, particularly in school settings, is important because of the high number of contacts within this subpopulation. Measles transmission in the school environment has been confirmed in a number of studies [35, 36]. When looking at the between-age group transmission, we observed that a substantial number of measles cases in older age strata, especially in the adult group (57.6%), was sourced from the 0–4 years group. The projected contact matrix implies that mixing rates are high between this age group and the age group of their parents and that it is likely that these parents have been exposed to measles and acquire infection from their children. Clustering of disease susceptibility within households can boost the likelihood and the persistence of disease outbreaks [9, 37]. Therefore, we recommend more in-depth analyses on measles transmission in high-contact settings (e.g. households and schools) in future work.

Our study provided insights into the spatial interaction between different geographical units in disease transmission. We found an agglomeration effect that measles incidence in the epidemic component scaled (slower) with the population size of the ‘importing’ age stratum *g* in province *r*. Since long-distance human movement has an important role in disease diffusion, applying the age-dependent power law formulation was appropriate in shaping the spatial interaction across age strata in relation to different neighbourhood orders [25, 38]. We observed that in the 0–4 years group, the power law puts more weights on local and first-order neighbour transmissions than in other age groups, which experienced the faster distance decay of transmission from the nearest neighbour. This implied that infections in older age groups were more likely to happen within their (provincial) home residence whereas cases in young children possibly depended on past cases from the same or neighbouring provinces. Although power law approximation is helpful to investigate disease spread [38], network data (e.g. local road, air data) could be conceivably taken into account as they could be a good proxy for the stochastic human transportation. Unfortunately, we did not have such data available for our study. We suggest that future research addresses this gap to yield further understanding in the spatial disease spreading.

The high number of infected persons among young children suggests that continuation of the routine two-dose vaccination programme for this group is critical. Furthermore, catch-up campaigns at the local rather than national level should be considered, and the focus should be on older age groups. This serves not only to improve local vaccination coverage across a broader age spectrum but also to reduce the risk that susceptible individuals, who may group together in schools and households, for example, may become infected through transmission from other age groups. We also suggest that health communication should receive more attention in future control measures, such as caution for school children and parents for better prevention of measles transmission in those environments.

We recorded several limitations in our study. First, our model was restricted with an autoregression on cases at previous time *t* − 1 but neglected cases at larger lags, which may improve the model fit [39]. However, our sensitivity analysis using weekly counts, which may better reflect the serial interval of measles, resulted in similar conclusions. Second, we relied on the number of contacts between strata calculated from a survey in Northern Vietnam anno 2007; this may not reflect the ‘true’ contact patterns in the Southern region. When more social contact data become available, we could also take into account the characteristics of contact networks in space (e.g. location of contact) and time (e.g. duration of contacts) as they may provide more information in the spatial disease spread [40]. Besides, higher reporting rates in children may explain the prevailing number of infections in younger age groups. Our model may, thus, underestimate the incidence in younger children and overestimate the incidence in older groups. The impact of such underreporting on the endemic–epidemic model is an important topic of future research. Finally, we did not consider local vaccination coverage and how immunity levels vary, for example because of previous outbreaks, as model inputs to infer the level of susceptibility. This is certainly a topic of interest when sufficient data are available.

In summary, we used an age-structured endemic–epidemic model of infectious disease counts to have insights into the transmission dynamics of measles in Southern Vietnam, based on measles surveillance data. In young children, within-age-group transmission was dominant whereas between-age-group transmission had stronger effects among older age groups. Furthermore, local and first-order neighbour transmission played a critical role in the diffusion of the disease despite age groups. Our study findings could be useful for age-targeted measles control in future as it gives insights into high-risk subgroups and key factors that are critical to the transmission dynamics (e.g. contacts between age groups, spatial interaction).

## Data Availability

Raw data were generated at the Pasteur Institute in Ho Chi Minh City, Vietnam. Derived data supporting and codes for reproducibility of the findings of this study are available at https://github.com/trangnguyenpmd/measlesVietnam_agestratified_model.

## Supplementary material

For supplementary material accompanying this paper visit http://doi.org/10.1017/S0950268822001431. click here to view supplementary material
