# Supplementary material for: Measles epidemic in Southern Vietnam: an age-stratified spatio-temporal model for infectious disease counts
Source: Epidemiol Infect. 2022 Sep 12;150:e169. doi: 10.1017/S0950268822001431 (PMC9980966; doi:10.1017/S0950268822001431)
Supplement: Supplementary file 1 [file S0950268822001431sup001.zip › S0950268822001431sup006.docx]

**Supplementary table**

Supplementary Table S1: Sensitivity analysis of age-stratified spatio-temporal models for surveillance data of measles in Southern Vietnam. The first three columns list the fitted models corresponding to different assumptions on spatial transmission weights, the age-structured contact matrices, and the resolution of the data used.

| Model with assumption of | | | Log-likelihood | Akaike Information Criterion | The power adjustment $\kappa$ of the contact matrix (95% CI) |
| --- | --- | --- | --- | --- | --- |
| Spatial structure | Contact matrix* | Measles data resolution |  |  |  |
| Power law (all age groups) | Projected contact matrix $\boldsymbol{C}_{\boldsymbol{(P)}}$ | weekly | -10,231 | 20,541 | --- |
|  | Adjusted contact matrix $\boldsymbol{C}_{\boldsymbol{(P)}}^{\boldsymbol{\kappa}}$ | weekly | -10,170 | 20,421 | 0.18 (0.13-0.24) |
|  | Original contact matrix $\boldsymbol{C}$ | daily | -29,803 | 59,685 | --- |
|  | Adjusted contact matrix $\boldsymbol{C}^{\boldsymbol{\kappa}}$ | daily | -29,795 | 59,671 | 1.33 (1.18-1.51) |
|  | Contact matrix $\boldsymbol{C}_{\boldsymbol{rate (2009)}}$ | daily | -29,772 | 59,622 | --- |
|  | Adjusted contact matrix $\boldsymbol{C}_{\boldsymbol{rate (2009)}}^{\boldsymbol{\kappa}}$ | daily | -29,768 | 59,617 | 0.79 (0.67-0.94) |
|  | Contact matrix $\boldsymbol{C}_{\boldsymbol{rate}\boldsymbol{(}\boldsymbol{2019}\boldsymbol{)}}$ | daily | -29,777 | 59,632 | --- |
|  | Adjusted contact matrix $\boldsymbol{C}_{\boldsymbol{rate (2019)}}^{\boldsymbol{\kappa}}$ | daily | -29,771 | 59,625 | 0.76 (0.64-0.90) |
| Group-specific power law | Projected contact matrix $\boldsymbol{C}_{\boldsymbol{(P)}}$ | weekly | -10,215 | 20,515 | --- |
|  | Adjusted contact matrix $\boldsymbol{C}_{\boldsymbol{(P)}}^{\boldsymbol{\kappa}}$ | weekly | -10,156 | 20,399 | 0.16 (0.11-0.22) |
|  | Original contact matrix $\boldsymbol{C}$ | daily | -29,747 | 59,578 | --- |
|  | Adjusted contact matrix $\boldsymbol{C}^{\boldsymbol{\kappa}}$ | daily | -29,473 | 59,573 | 1.16 (1.04-1.29) |
|  | Contact matrix $\boldsymbol{C}_{\boldsymbol{rate (2009)}}^{\boldsymbol{\kappa}}$ | daily | -29,724 | 59,532 | --- |
|  | Adjusted contact matrix $\boldsymbol{C}_{\boldsymbol{rate (2009)}}^{\boldsymbol{\kappa}}$ | daily | -29,723 | 59,533 | 0.92 (0.80-1.06) |
|  | Contact matrix $\boldsymbol{C}_{\boldsymbol{rate}\boldsymbol{(}\boldsymbol{2019}\boldsymbol{)}}$ | daily | -29,727 | 59,539 | --- |
|  | Adjusted contact matrix $\boldsymbol{C}_{\boldsymbol{rate (2019)}}^{\boldsymbol{\kappa}}$ | daily | -29,726 | 59,539 | 0.91 (0.79-1.04) |

***** The projected contact matrix $\boldsymbol{C}_{\boldsymbol{(P)}}$ is calculated from the original contact matrix $\boldsymbol{C}$ using density correction method in Arregui et al (2018) [22]. The per capital contact matrix $\boldsymbol{C}_{\boldsymbol{(rate)}}$ that each element represents the per capital contact rate between age strata and was divided by the Vietnamese age-structured population size in 2009 and in 2019 (the census data) [20].
