# Supplementary figures and images for: Measles epidemic in Southern Vietnam: an age-stratified spatio-temporal model for infectious disease counts
Source: Epidemiol Infect. 2022 Sep 12;150:e169. doi: 10.1017/S0950268822001431 (PMC9980966; doi:10.1017/S0950268822001431)

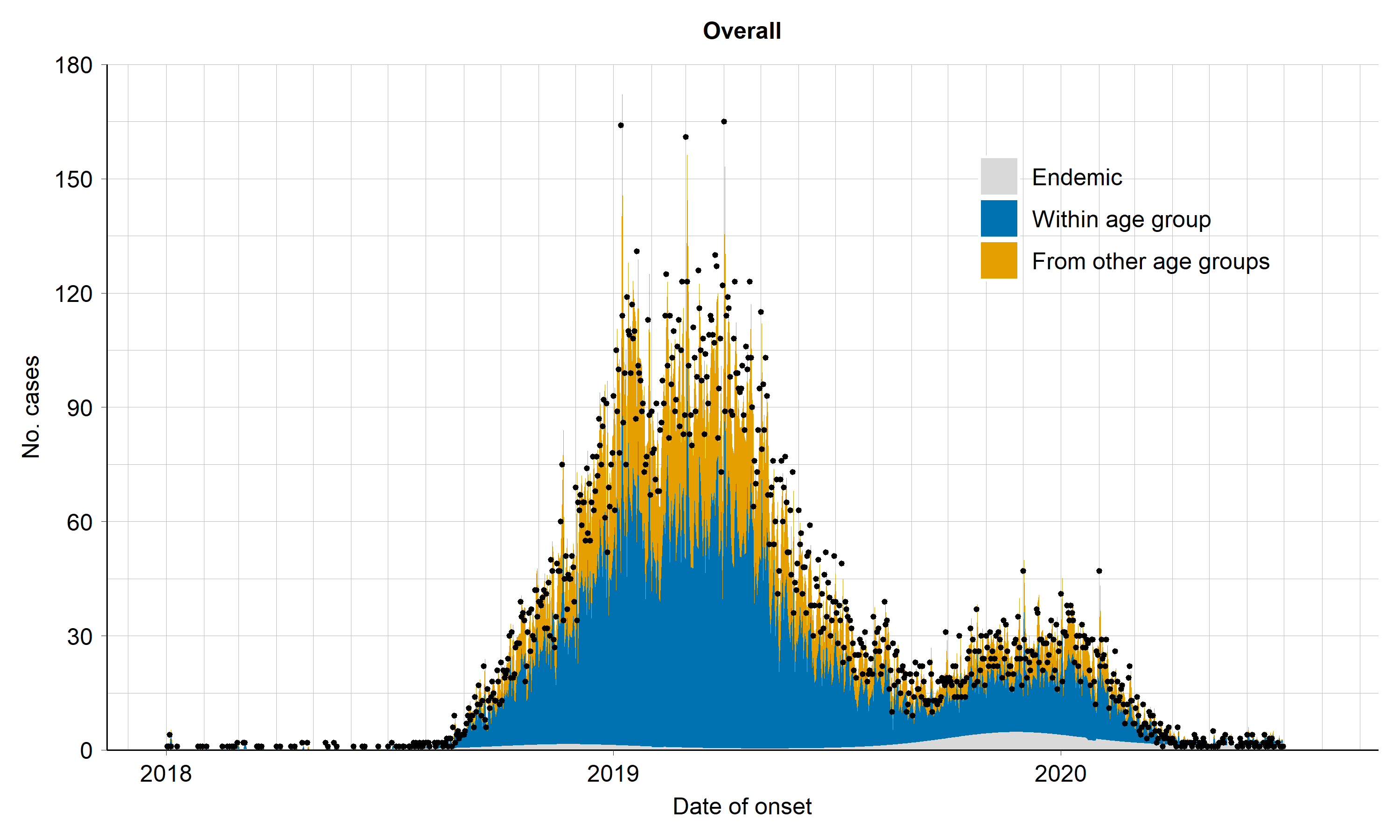

Supplement: Supplementary file 1 [file S0950268822001431sup001.zip › S0950268822001431sup001.tif]

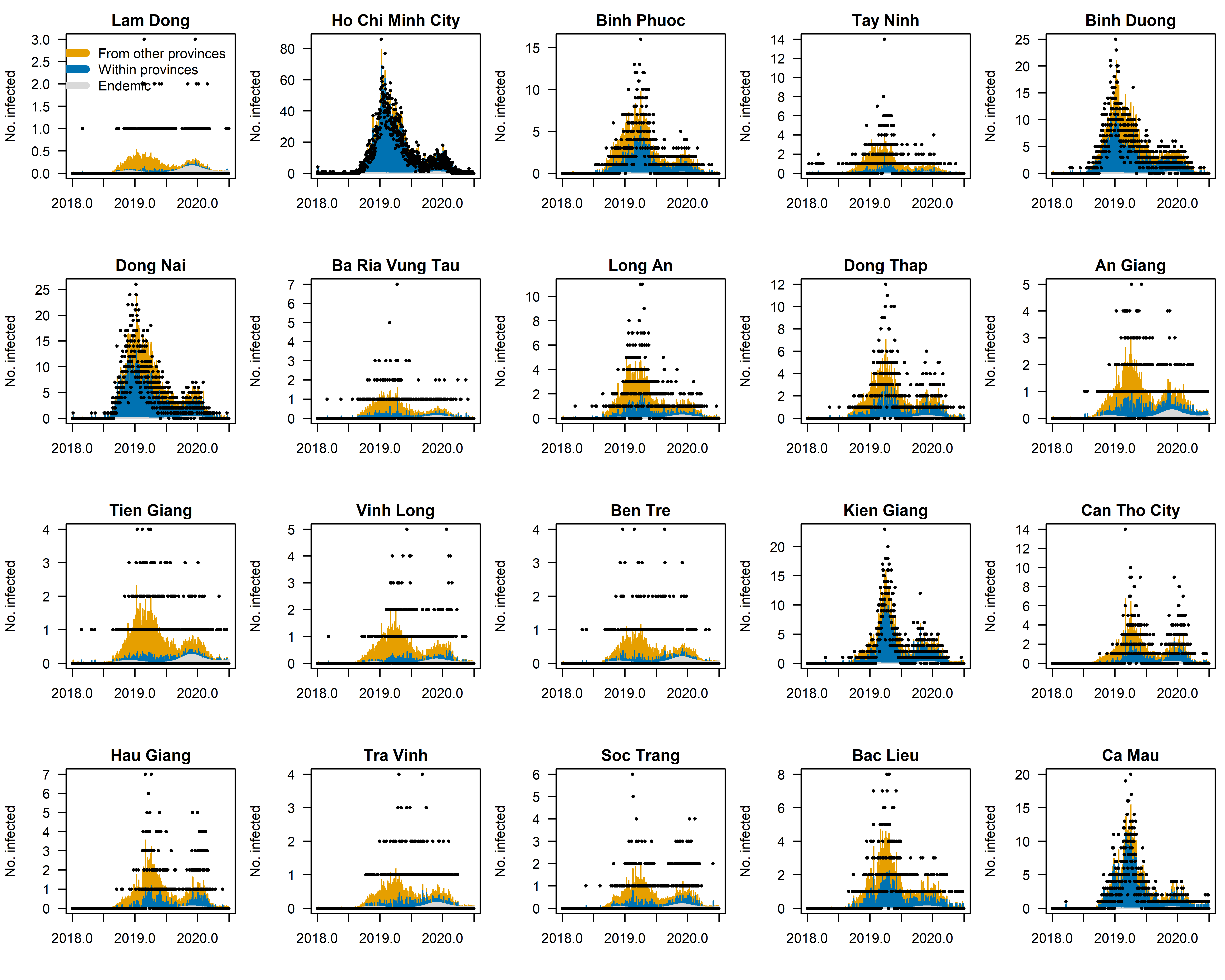

Supplement: Supplementary file 1 [file S0950268822001431sup001.zip › S0950268822001431sup002.tiff]

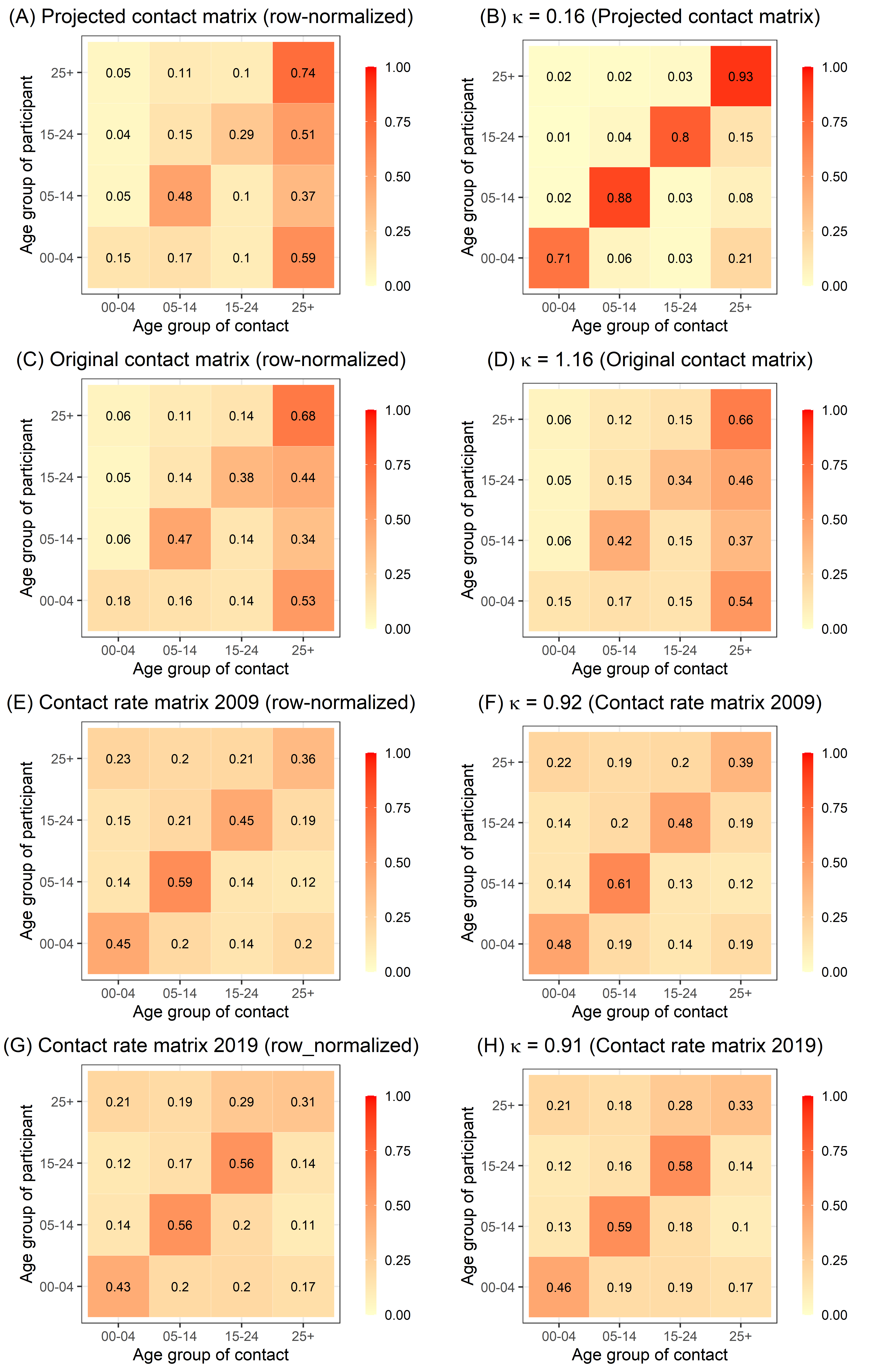

Supplement: Supplementary file 1 [file S0950268822001431sup001.zip › S0950268822001431sup003.tiff]

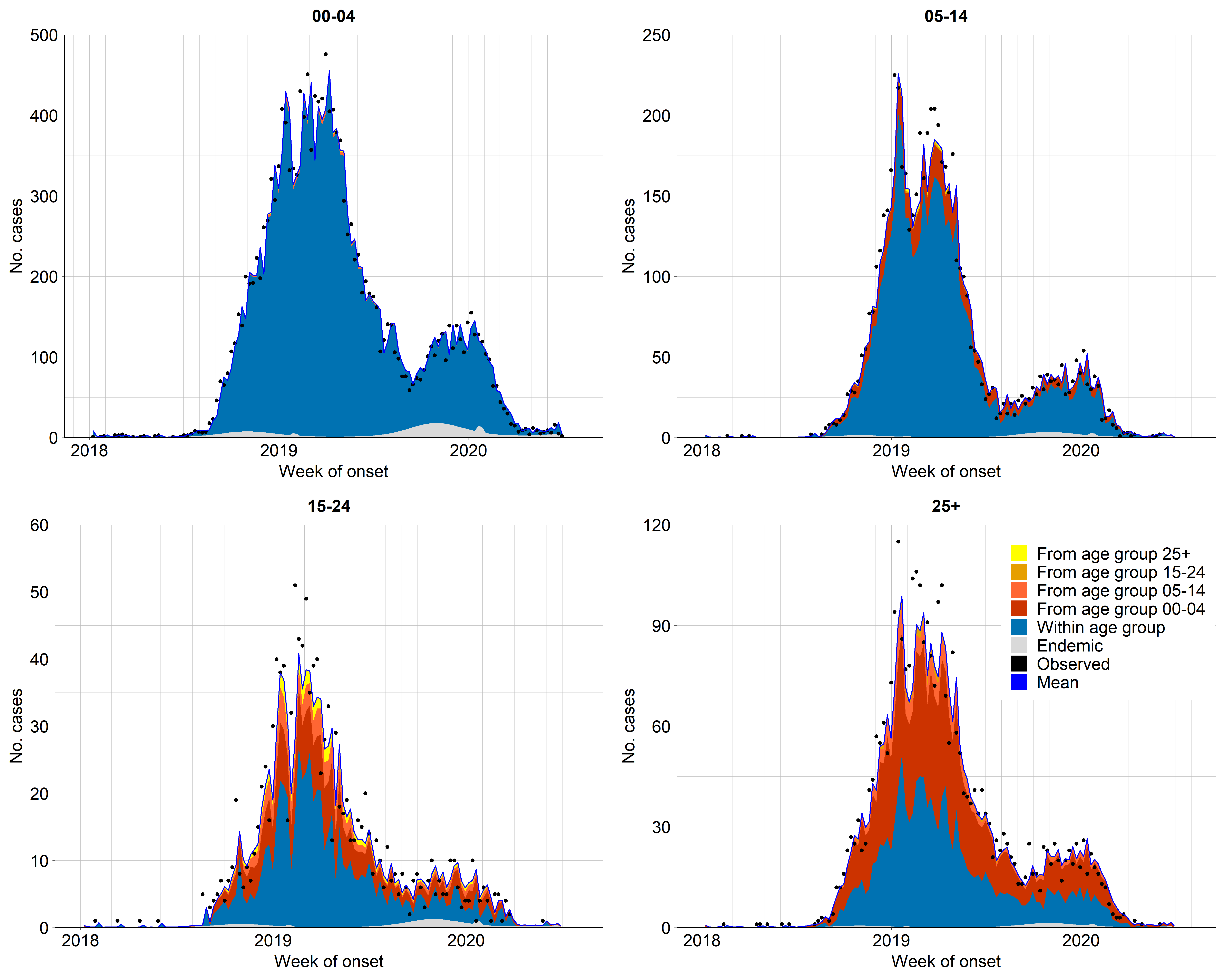

Supplement: Supplementary file 1 [file S0950268822001431sup001.zip › S0950268822001431sup004.tiff]

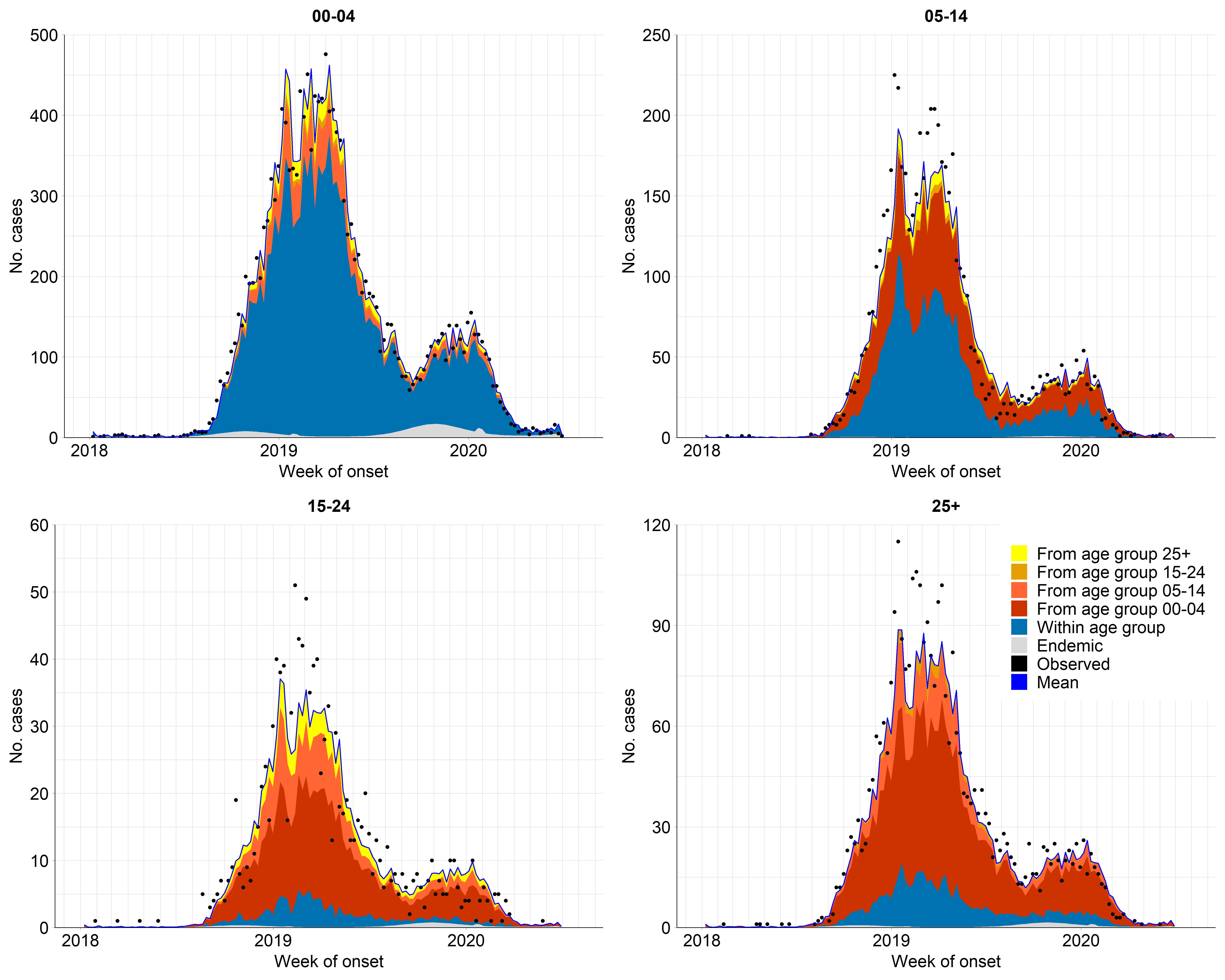

Supplement: Supplementary file 1 [file S0950268822001431sup001.zip › S0950268822001431sup005.tiff]
